# Correction: Investigating ethical tradeoffs in crisis standards of care through simulation of ventilator allocation protocols

**DOI:** 10.1371/journal.pone.0315138

**Published:** 2024-12-03

**Authors:** Jonathan Herington, Jessica Shand, Jeanne Holden-Wiltse, Anthony Corbett, Richard Dees, Chin-Lin Ching, Margie Shaw, Xueya Cai, Martin Zand

The Figs 1–4 are uploaded incorrectly. Please see the correct Figs 1–4 here.

**Fig 1:**
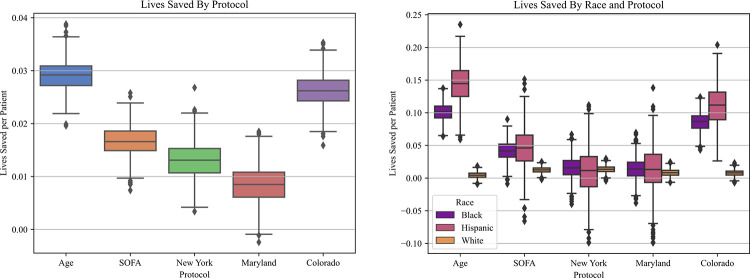
Lives saved per patient by protocol.

**A**. Overall increase in lives saved per patient, for each protocol, at 50% scarcity. **B**. Lives saved per patient by race/ethnicity, for each protocol, at 50% scarcity.

**Fig 2:**
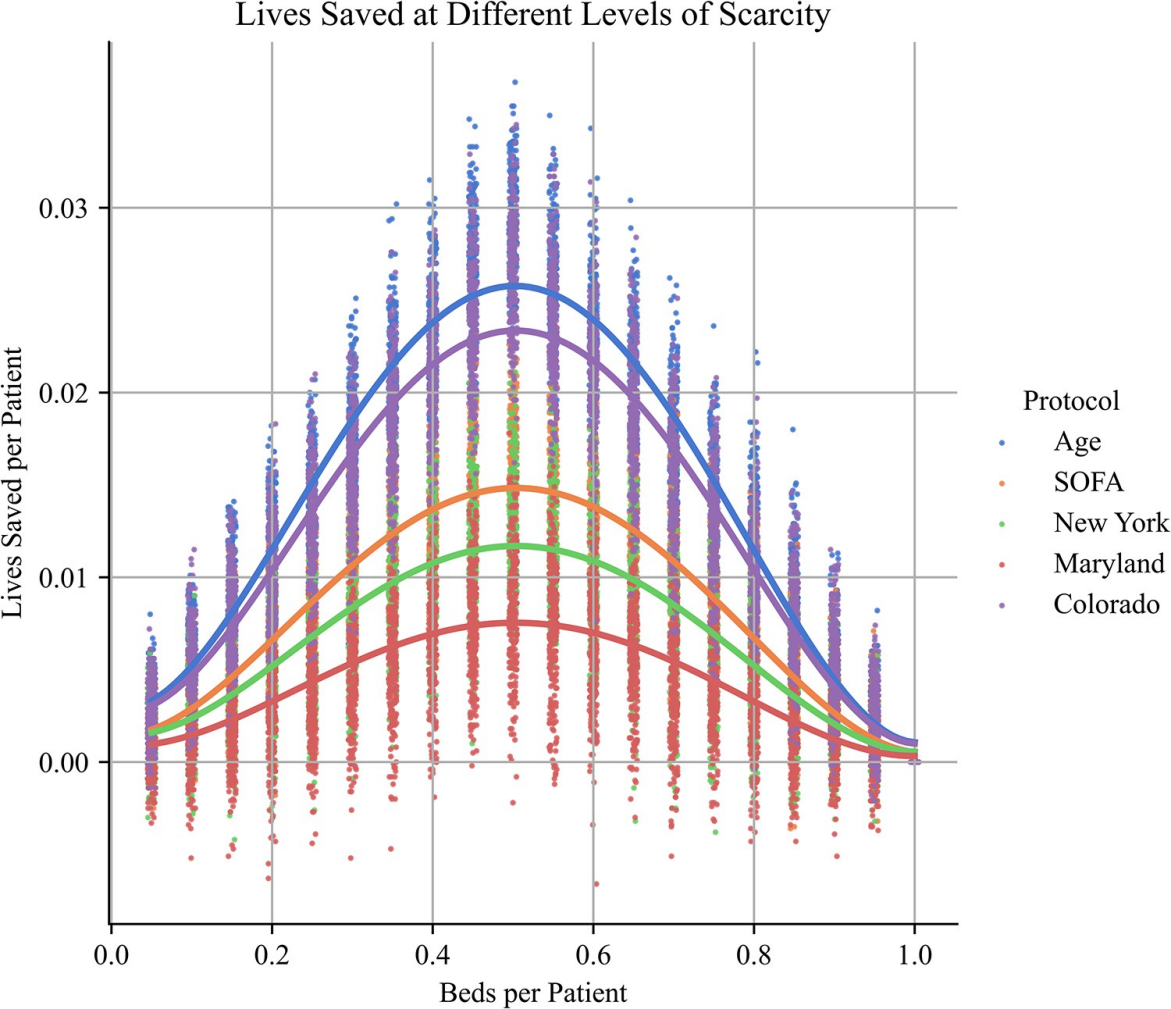
Lives saved at different levels of scarcity, for each protocol.

The greatest differences between protocols occur at moderate levels of scarcity (i.e. ~0.5 beds per patient), and differences between protocols decline at both high and low levels of scarcity.

**Fig 3:**
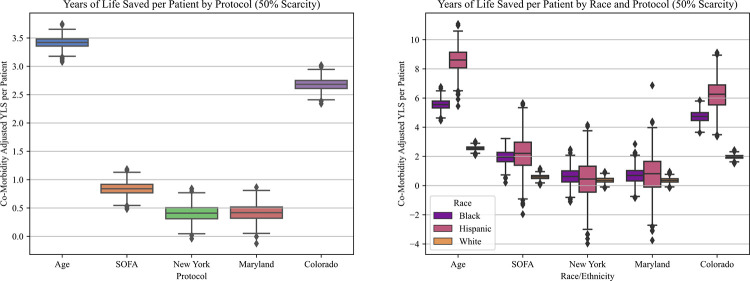
Life years saved per patient.

**A**. Overall increase in life years saved per patient, for each protocol, at 50% scarcity. **B**. Life years saved per patient by racial group, for each protocol, at 50% scarcity.

**Fig 4:**
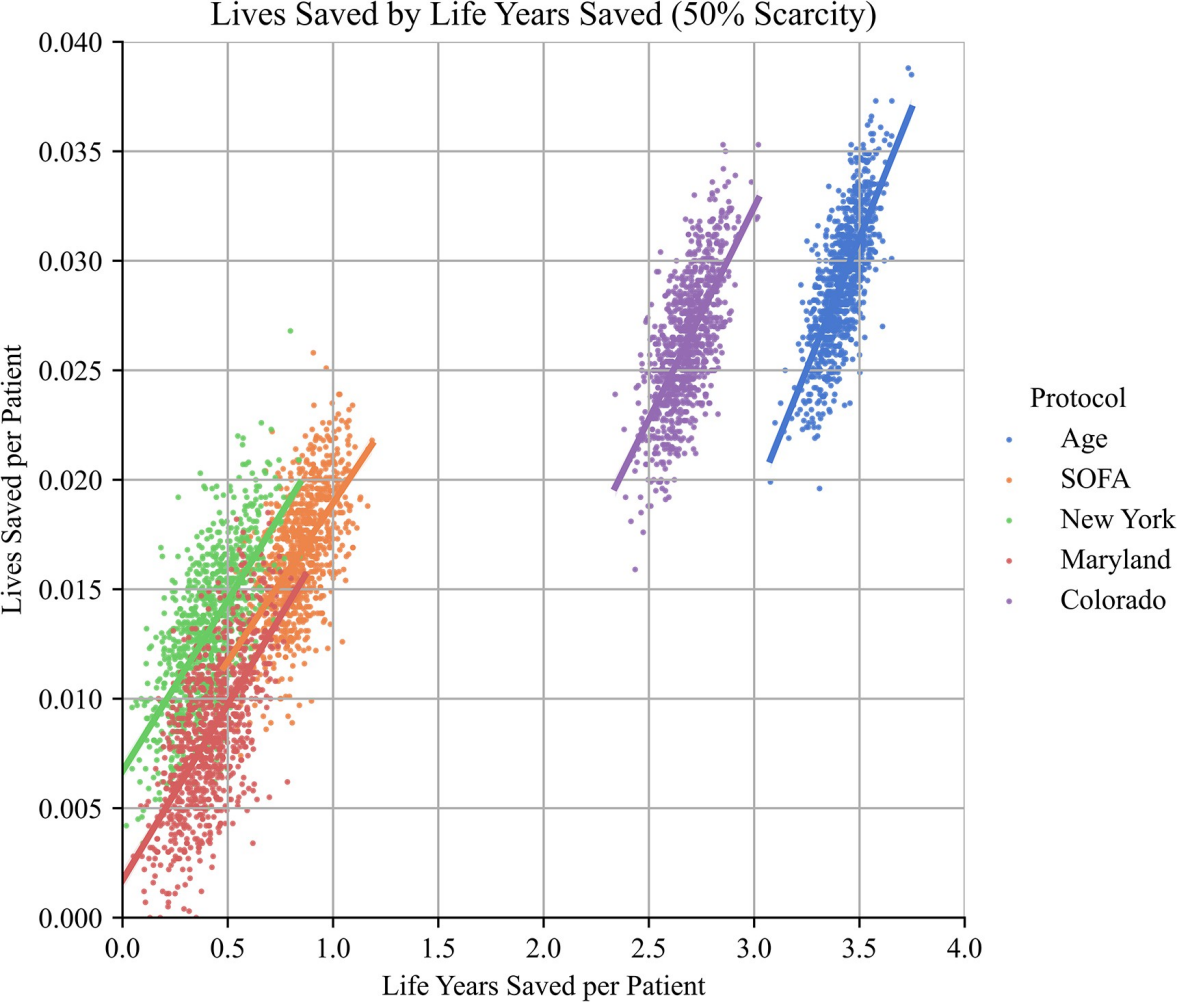
Tradeoffs between life years saved and lives saved.

While there is significant variance in numbers of lives and life years saved, there is a strong positive correlation between each statistic for all protocols.
