# Supplementary material for: Investigating ethical tradeoffs in crisis standards of care through simulation of ventilator allocation protocols
Source: PLoS One. 2024 Sep 12;19(9):e0300951. doi: 10.1371/journal.pone.0300951 (PMC11392394; doi:10.1371/journal.pone.0300951)
Supplement: S1 Appendix — Tabular summary of simulated protocols. (DOCX) [file pone.0300951.s001.docx]

## S1 Appendix. Crisis Standards of Care Protocols

Herington et al. (2024) “Investigating Ethical Tradeoffs in Crisis Standards of Care through Simulation of Ventilator Allocation Protocols”

**Table S1 A: Summary of Crisis Standard of Care Protocols.**

| **Protocol** | **Score Part A** | **Score Part B** | **Ranking Criteria** |
| --- | --- | --- | --- |
| **Lottery** | Assign randomly generated number between 0 and 5000 |  | Rank by Score A |
| **Age Band** | Age <25 1 pts  25-34 2 pts  35-44 3 pts  45-54 4 pts  55-64 5 pts  65-74 6 pts  75-84 7 pts  ≥85 8 pts |  | Rank by Score A, ties broken by lottery |
| **Pure SOFA** | Max 24hr SOFA score |  | Rank by Score A, ties broken by lottery |
| **New York ‘15** | SOFA ≤7 1 pts  SOFA 8-11 2 pts  SOFA ≥12 3 pts |  | Rank by Score A, ties broken by lottery |
| **Maryland ‘21** | SOFA ≤8 1 pts  SOFA 9-11 2 pts  SOFA 12-14 3 pts  SOFA ≥15 4 pts | Elixhauser <12 0 pts  Elixhauser ≥12 3 pts | Rank by rank by sum of Score A + Score B, ties broken by lottery |
| **Colorado ‘20** | SOFA 0 0 pts  SOFA 1-5 1 pts  SOFA 6-9 2 pts  SOFA 10-12 3 pts  SOFA ≥13 4 pts | mCCI 0 0 pts  mCCI 1-2 1 pts  mCCI 3-5 2 pts  mCCI 6-7 3 pts  mCCI ≥8 4 pts | Rank by sum of Score A + Score B, ties broken by lottery |

**Table S1 B: modified Charlson Comorbidity Index (mCCI)** developed for Colorado protocol (6). mCCI is calculated as sum of points for each variable present upon admission.

| Variable | Points | Variable | Points |
| --- | --- | --- | --- |
| Age  <50  50-59  60-69  70-79  ≥80  Chronic Heart Failure  Dementia  Chronic Pulmonary Disease | +0  +1  +2  +3  +4  +2  +2  +1 | Connective Tissue Disease  Liver Disease (Mild)  Liver Diseases (Moderate or Severe)  Diabetes Mellitus with Chronic Complications  Hemiplegia/Paraplegia due to CVA  Renal Disease  Metastatic Solid Tumor  Any active malignancy including leukemia/lymphoma  AIDS | +1  +2  +4  +1  +2  +1  +6  +2  +4 |
