# Supplementary material for: Investigating ethical tradeoffs in crisis standards of care through simulation of ventilator allocation protocols
Source: PLoS One. 2024 Sep 12;19(9):e0300951. doi: 10.1371/journal.pone.0300951 (PMC11392394; doi:10.1371/journal.pone.0300951)
Supplement: S2 Appendix — (DOCX) [file pone.0300951.s002.docx]

## S2 Appendix. Characteristics for Included and Excluded Encounters.

Herington et al. (2024) “Investigating Ethical Tradeoffs in Crisis Standards of Care through Simulation of Ventilator Allocation Protocols”

| **Table S2 A: Descriptive Statistics for INCLUDED and EXCLUDED Encounters**, April 2020 to May 2021**.** | | | | | | | | |
| --- | --- | --- | --- | --- | --- | --- | --- | --- |
|  | **Included** | | **Excluded** | | **Survival**% (CI) | | **Age-Adjusted Survival** *% (CI)* | |
| **Feature** | **n** | **%** | **n** | **%** | **Included** | **Excluded** | **Included** | **Excluded** |
| **Overall** | **3707** |  | **898** |  | **72 (*71-74*)** | **72 (69-76)** | **N/A** | **N/A** |
| **Sex** |  |  |  |  |  |  |  |  |
| Male | 2197 | 59 | 520 | 58 | 73 (*71 - 75)* | 71 (66-76) | 84 *(80 - 87)* | 84 (77-89) |
| Female | 1510 | 41 | 378 | 42 | 72 (*69 - 74*) | 74 (68-79) | 84 *(80 - 86)* | 83 (64-91) |
|  |  |  |  |  |  |  |  |  |
| **Age** |  |  |  |  |  |  |  |  |
| <25 | 104 | 3 | 30 | 3 | 93 (*86 - 97*) | 93 (76-99) |  |  |
| 25-34 | 235 | 6 | 46 | 5 | 90 (*85 - 93*) | 89 (75-96) |  |  |
| 35-44 | 247 | 7 | 67 | 7 | 83 (*77 - 87*) | 87 (75-94) |  |  |
| 45-54 | 456 | 12 | 141 | 16 | 81 (*77 - 85*) | 83 (75-89) |  |  |
| 55-64 | 868 | 23 | 223 | 25 | 76 (*73 - 79*) | 74 (66-80) |  |  |
| 65-74 | 980 | 26 | 218 | 24 | 69 (*66* - *73*) | 63 (54-70) |  |  |
| 75-84 | 629 | 17 | 128 | 14 | 60 (*54 - 64*) | 59 (47-70) |  |  |
| >85 | 188 | 5 | 41 | 5 | 50 (*38 - 59*) | 56 (31-74) |  |  |
|  |  |  |  |  |  |  |  |  |
| **Race** |  |  |  |  |  |  |  |  |
| AAPI, non-Hispanic | 50 | 1 | 11 | 1 | 78 (*61 - 89*) | 64 (7-90) | 87 *(24 - 97)* | 70 (3-95) |
| AIAN, non-Hispanic | 3 | >1 | 1 | >1 | 100 | 100 | 100 | 100 |
| Black, non-Hispanic | 577 | 16 | 197 | 22 | 76 (*72 - 80*) | 76 (68-82) | 82 *(74 - 87)* | 81 (43-94) |
| Hispanic | 152 | 4 | 40 | 4 | 82 (*73 - 88*) | 75 (54-88) | 89 *(78 - 94)* | 83 (62-93) |
| White, non-Hispanic | 2752 | 74 | 606 | 67 | 71 (*69 - 74*) | 72 (67-76) | 85 *(81 - 87)* | 87 (78-90) |
| >1 Race, non-Hispanic | 16 | >1 | 8 | 1 | 75 (*36 - 93*) | 100 | 87 *(23 - 96)* | 100 |
| Unknown, non-Hispanic | 157 | 4 | 35 | 4 |  |  | 79 *(81 - 87)* | 42 (0-90) |
|  |  |  |  |  |  |  |  |  |
| **COVID Status** |  |  |  |  |  |  |  |  |
| Negative | 2431 | 66 | 431 | 48 | 73 (*71 - 75*) | 71 (65-76) | 84 *(81 - 86)* | 82 (72-87) |
| Positive | 1276 | 34 | 429 | 48 | 71 (*68 - 74)* | 75 (69-79) | 83 (*76 - 88)* | 90 (78-92) |
| Unknown | - | - | 38 | 4 | - | 61 (35-78) | - | 85 (0-92) |
|  |  |  |  |  |  |  |  |  |

**Table S2 B: Distribution of SOFA Score**


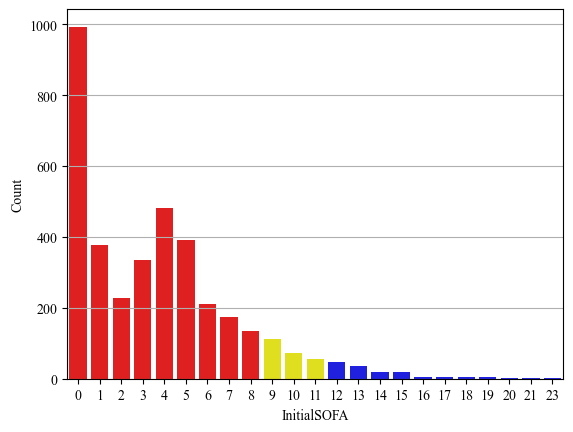


**Fig S2 B: Histogram of SOFA score distribution.**


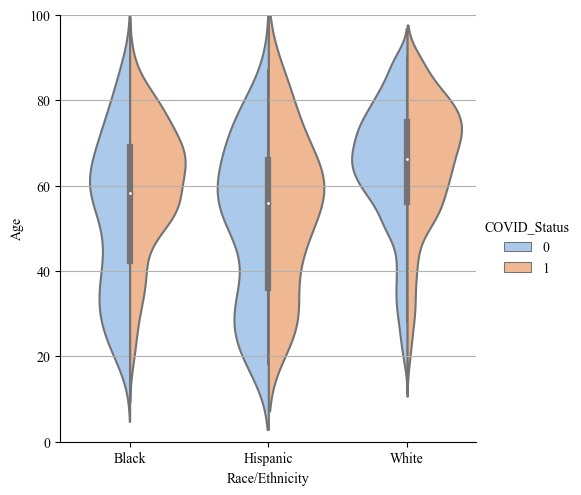


**Fig S2 A:** **Violin plot of age distribution** by race and ethnicity and COVID-positivity status (AAPI, AIAN, Multi-Racial and Unknown sub-populations are not shown).

| **SOFA** | **n** | **%** |
| --- | --- | --- |
| 0 | 993 | 26.79 |
| 1 | 378 | 10.20 |
| 2 | 227 | 6.12 |
| 3 | 334 | 9.01 |
| 4 | 482 | 13.00 |
| 5 | 392 | 10.57 |
| 6 | 211 | 5.69 |
| 7 | 175 | 4.72 |
| 8 | 134 | 3.61 |
| 9 | 111 | 2.99 |
| 10 | 72 | 1.94 |
| 11 | 55 | 1.48 |
| 12 | 48 | 1.29 |
| 13 | 36 | 0.97 |
| 14 | 19 | 0.51 |
| 15 | 20 | 0.54 |
| 16 | 4 | 0.11 |
| 17 | 4 | 0.11 |
| 18 | 5 | 0.13 |
| 19 | 4 | 0.11 |
| 20 | 1 | 0.03 |
| 21 | 1 | 0.03 |
| 22 | 0 | 0.00 |
| 23 | 1 | 0.03 |
| 24 | 0 | 0.00 |

**Table S2 C: Patient characteristics across race/ethnicity.**

| **Statistic** | **Non-Hispanic, Black** | **Hispanic, All Races** | **Non-Hispanic, White** | **Overall** |
| --- | --- | --- | --- | --- |
| Male (%) | 52.86 | 61.84 | 60.28 | 59.27 |
| Age (µ) | 56.03 | 52.72 | 64.19 | 62.09 |
| COVID (% pos) | 44.89 | 39.47 | 32.63 | 34.42 |
| Charlson (µ) | 0.44 | 0.45 | 0.56 | 0.54 |
| Elixhauser (µ) | 2.63 | 2.21 | 2.96 | 2.88 |
| SOFA Score (µ) | 3.46 | 3.14 | 3.68 | 3.66 |
| LE, NVSS (µ years) | 23.66 | 29.46 | 20.15 | 21.40 |
| LE, Cho (µ years) | 27.37 | 32.97 | 23.25 | 24.62 |

**Table S2 D: Top 15 Comorbidities in our COVID negative sample.** Acute ICD-10 codes for our COVID negative population were associated with the relevant AHRQ (Agency for Healthcare Research and Quality) CCSR (Clinical Classifications Software Refined) category to provide a general overview of the conditions which precipitated mechanical ventilation in the absence of COVID.

| **AHRQ Clinical Classifications Category Description** | **Count** | **% of Codes** |
| --- | --- | --- |
| Respiratory signs and symptoms | 412 | 12.4% |
| Nervous system signs and symptoms | 194 | 5.9% |
| Cardiac arrest and ventricular fibrillation | 152 | 4.6% |
| Abnormal findings without diagnosis | 140 | 4.2% |
| Shock | 133 | 4.0% |
| Septicemia | 129 | 3.9% |
| Respiratory failure; insufficiency; arrest | 122 | 3.7% |
| Fluid and electrolyte disorders | 106 | 3.2% |
| Nonspecific chest pain | 100 | 3.0% |
| Circulatory signs and symptoms | 93 | 2.8% |
| Acute and unspecified renal failure | 82 | 2.5% |
| Pericarditis and pericardial disease | 72 | 2.2% |
| Other unspecified injury | 67 | 2.0% |
| Cardiac dysrhythmias | 58 | 1.7% |
| Abdominal pain and other digestive/abdomen signs and symptoms | 57 | 1.7% |
| … | … | … |
| **TOTAL** | **2430** | **100%** |
