# Supplementary material for: Investigating ethical tradeoffs in crisis standards of care through simulation of ventilator allocation protocols
Source: PLoS One. 2024 Sep 12;19(9):e0300951. doi: 10.1371/journal.pone.0300951 (PMC11392394; doi:10.1371/journal.pone.0300951)
Supplement: S3 Appendix — (DOCX) [file pone.0300951.s003.docx]

## S3 Appendix. Association between Survival, SOFA Score and Age

Herington et al. (2024) “Investigating Ethical Tradeoffs in Crisis Standards of Care through Simulation of Ventilator Allocation Protocols”


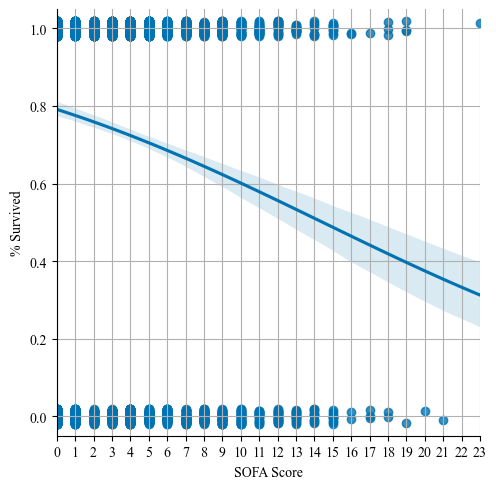


**Fig S3 A:**  **Survival rates by initial SOFA score and age**. Single explanatory variable logistic regressions and 95% CI plotted. **(I)** In these simple univariable logit models (Survival ~ SOFA” and “Survival ~Age”), each SOFA score point at intubation is associated with a 9.25% (7.38 – 11.09%) decrease in the probability of survival to discharge (Logit coeff. = -0.0971, std err = 0.010). **(II)** In our population, each year of subject age is associated with a 3.65% (3.11 – 4.18%) decrease in the probability of survival to discharge (Logit coeff. = -0.0372, std err = 0.003).


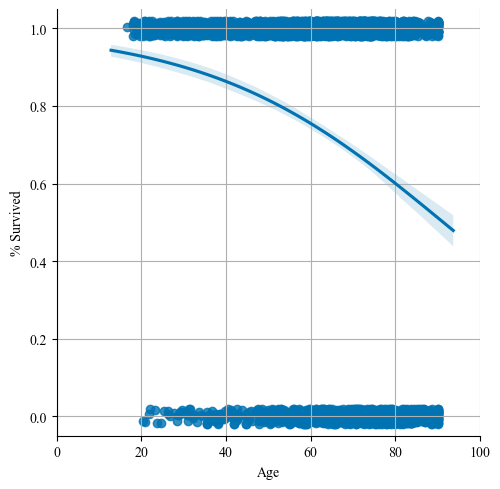


The formula for the regression plotted in Figure S3 A (I), Logit("Survived ~ SOFA") produces the following odds ratios:

|  | **Odds Ratio** | **95% Lower CI** | **95% Upper CI** |
| --- | --- | --- | --- |
| **Intercept** | 3.791854 | 3.400778 | 4.227903 |
| **SOFA Score** | 0.911925 | 0.894227 | 0.929974 |

------

The formula for the regression plotted in Figure S3 A (II), Logit("Survived ~ Age") produces the following output:

|  | **Odds Ratio** | **95% Lower CI** | **95% Upper CI** |
| --- | --- | --- | --- |
| **Intercept** | 26.547393 | 18.613299 | 37.863470 |
| **Age** | 0.964740 | 0.959699 | 0.969808 |


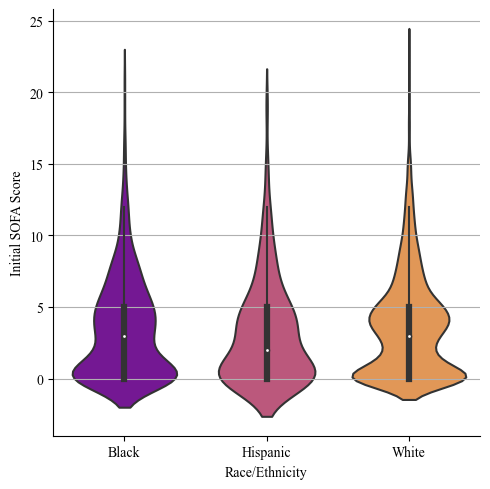

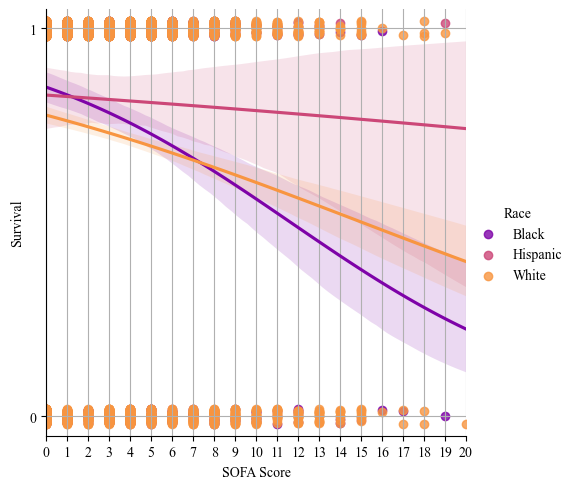


**Fig S3 B: Initial SOFA Score and survival by race/ethnicity. A**. Distribution of SOFA score by race/ethnicity **B.** In a single explanatory variable logistic regression analyzing the relationship between SOFA score and survival for each racial/ethnic sub-population, each SOFA score point at intubation is associated with decreases in the probability of survival to discharge of 13.15% for non-Hispanic, Black patients, 3.29% (-13.2% to +8.55%) for Hispanic patients of any race, and 8.71% for non-Hispanic, White patients. Logistic regressions and 95% CI plotted.

The formula for each of the regressions plotted in Figure S3 B (II) Logit("Survived ~ SOFA Score"). For each sub-population it produces the following output:

**Black sub-population regression**

==============================================================================

coef std err z P>|z| [0.025 0.975]

-------------------------------------------------------------------------------

Intercept 1.7227 0.152 11.353 0.000 1.425 2.020

InitialSOFA -0.1480 0.027 -5.489 0.000 -0.201 -0.095

===============================================

**Hispanic sub-population**

==============================================================================

coef std err z P>|z| [0.025 0.975]

-------------------------------------------------------------------------------

Intercept 1.5722 0.281 5.587 0.000 1.021 2.124

InitialSOFA -0.0259 0.056 -0.462 0.644 -0.136 0.084

==============================================================================

**White sub-population regression**

==============================================================================

coef std err z P>|z| [0.025 0.975]

-------------------------------------------------------------------------------

Intercept 1.2443 0.064 19.570 0.000 1.120 1.369

InitialSOFA -0.0827 0.012 -7.154 0.000 -0.105 -0.060

==============================================================================

**Table S3 A: Odds ratio of survival for whole population and racial sub-populations.** A logistic regression model was used to calculate the relationship between survival and six explanatory variables: race (ref: White, non-Hispanic), sex-assigned-at-birth (ref: female), SOFA score, Age, COVID positivity (ref:neg) and Elixhauser comorbidity index. The model was also run for each racial/ethnic sub-population. Odds-ratio are reported alongside 95% confidence intervals, significant effects (p=<0.01) in bold.

|  | **All** | | | **Black, non-Hispanic** | | | **Hispanic, Any race** | | | **White, non-Hispanic** | | |
| --- | --- | --- | --- | --- | --- | --- | --- | --- | --- | --- | --- | --- |
|  | **OR** | **[** | **]** | **OR** | **[** | **]** | **OR** | **[** | **]** | **OR** | **[** | **]** |
| **Black** | .980 | .784 | 1.226 |  |  |  |  |  |  |  |  |  |
| **Hispanic** | 1.219 | .787 | 1.889 |  |  |  |  |  |  |  |  |  |
| **COVID Pos** | .942 | .803 | 1.106 | 1.176 | .774 | 1.788 | 1.319 | .544 | 3.195 | 1.032 | .865 | 1.231 |
| **Male** | 1.043 | .894 | 1.217 | 1.035 | .687 | 1.557 | .954 | .843 | 1.079 | **.903** | .881 | .925 |
| **SOFA Score** | **.896** | **.877** | **.915** | **.855** | **.808** | **.905** | **.964** | .939 | .990 | **.963** | .956 | .969 |
| **Age** | **.964** | **.958** | **.969** | **.966** | **.953** | **.979** | 1.043 | .910 | 1.196 | **1.051** | 1.028 | 1.074 |
| **Elixhauser** | **1.054** | **1.034** | **1.075** | **1.069** | **1.012** | **1.129** | 1.319 | .544 | 3.195 | 1.032 | .865 | 1.231 |

The formula for the overall model using statsmodels logit function is:

logit( "Survived ~ SOFA + C(Race, Treatment(reference='White')) + Age + C(COVID_Status) + Sex + Elixhauser"

and produces the following output:

=======================================================================================

coef std err z P>|z| [0.025 0.975]

---------------------------------------------------------------------------------------

Intercept 3.6477 0.212 17.216 0.000 3.232 4.063

[T.>1 Race] -0.2996 0.614 -0.488 0.626 -1.503 0.904

[T.AAPI] 0.2580 0.358 0.721 0.471 -0.443 0.959

[T.AIAN] 12.0144 386.840 0.031 0.975 -746.177 770.206

[T.Black] -0.0200 0.114 -0.176 0.861 -0.244 0.204

[T.Hispanic] 0.1983 0.223 0.888 0.374 -0.239 0.636

[T.Unknown] -0.3745 0.183 -2.042 0.041 -0.734 -0.015

COVID_Status[T.1] -0.0595 0.082 -0.728 0.466 -0.220 0.101

Sex[T.Male] 0.0418 0.079 0.531 0.595 -0.112 0.196

InitialSOFA -0.1096 0.011 -10.149 0.000 -0.131 -0.088

Age -0.0371 0.003 -13.150 0.000 -0.043 -0.032

ECI_raw 0.0527 0.010 5.270 0.000 0.033 0.072
