# Supplementary material for: Investigating ethical tradeoffs in crisis standards of care through simulation of ventilator allocation protocols
Source: PLoS One. 2024 Sep 12;19(9):e0300951. doi: 10.1371/journal.pone.0300951 (PMC11392394; doi:10.1371/journal.pone.0300951)
Supplement: S4 Appendix — (DOCX) [file pone.0300951.s004.docx]

## S4 Appendix: Survival by Age and Protocol

Herington et al. (2024) “Investigating Ethical Tradeoffs in Crisis Standards of Care through Simulation of Ventilator Allocation Protocols”

**Fig S4 A:** **Survival rate by Age and Protocol.**


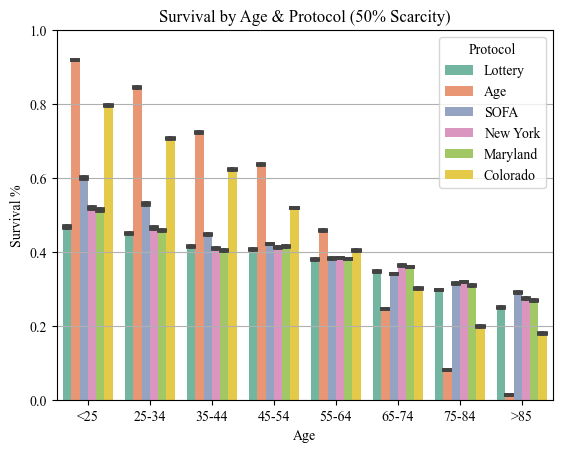


**Table S4 A: Survival by Age Group at 50% capacity.** Mean survival and 95% CI is reported for 1000 Monte Carlo simulations at the indicated level of scarcity.

| **Protocol** | **Mean Survival by Age-Group, %** | | | | | |
| --- | --- | --- | --- | --- | --- | --- |
|  | **Lottery** | **Age** | **Pure SOFA** | **New York, ‘15** | **Maryland, ‘21** | **Colorado, ‘20** |
| **<25** | 47 | 92 | 60 | 52 | 82 | 80 |
| **25-34** | 45 | 84 | 53 | 46 | 73 | 71 |
| **35-44** | 41 | 72 | 45 | 41 | 64 | 62 |
| **45-54** | 41 | 64 | 42 | 41 | 53 | 52 |
| **55-64** | 38 | 46 | 38 | 38 | 42 | 40 |
| **65-74** | 35 | 24 | 34 | 36 | 31 | 30 |
| **75-84** | 30 | 8 | 31 | 32 | 17 | 20 |
| **>85** | 25 | 1 | 29 | 27 | 8 | 18 |
