# Supplementary material for: Investigating ethical tradeoffs in crisis standards of care through simulation of ventilator allocation protocols
Source: PLoS One. 2024 Sep 12;19(9):e0300951. doi: 10.1371/journal.pone.0300951 (PMC11392394; doi:10.1371/journal.pone.0300951)
Supplement: S5 Appendix — (DOCX) [file pone.0300951.s005.docx]

## S5 Appendix. Capacity Sensitivity Analysis.

Herington et al. (2024) “Investigating Ethical Tradeoffs in Crisis Standards of Care through Simulation of Ventilator Allocation Protocols”

**Table S5 A: Survival by Protocol at different Capacities.** Mean survival and 95% CI is reported for 250 Monte Carlo simulations at the indicated level of scarcity. All findings within and across capacity-levels are statistically significant to P < 0.05.

| **Protocol** | **Mean Survival by Capacity (%)** | | | | |
| --- | --- | --- | --- | --- | --- |
|  | **20%** | **40%** | **60%** | **80%** | **90%** |
| **Lottery** | 14.52 (14.49-14.56) | 29.03 (28.99-29.07) | 43.53 (43.49-443.58) | 58.05 (58.02-58.09) | 65.30 (65.27-65.32) |
| **Age** | 15.67 (15.64-15.70) | 31.35 (31.31-31.39) | 45.85 (45.81-45.89) | 59.23 (59.19-59.27) | 65.86 (65.83-65.89) |
| **Pure SOFA** | 15.18 (15.14-15.22) | 30.35 (30.31-30.39) | 44.88 44.84-44.92 | 58.73 58.70-58.77 | 65.62 65.60-65.65 |
| **New York, ‘15** | 15.01 (14.98-15.05) | 30.08 (30.03-30.12) | 44.59 (44.55-44.64) | 58.57 (58.53-58.61) | 65.58 (65.55-65.61) |
| **Maryland, ‘21** | 14.85 (14.81-14.89) | 29.71 (29.67-29.75) | 44.21 (44.17-44.26) | 58.38 (58.35-58.42) | 65.46 (65.43-65.49 |
| **Colorado, ‘20** | 15.54 (15.51-15.57) | 31.14 (31.10-31.17) | 45.66 (45.62-45.70) | 59.10 (59.06-59.13) | 65.83 (65.80-65.86) |

**Table S5 B: Lives Saved by Protocol at different Capacities.** Mean survival and 95% CI is reported for 250 Monte Carlo simulations at the indicated level of scarcity. All findings within and across capacity-levels are statistically significant to P < 0.05.

| **Protocol** | **Lives Saved by Capacity (per thousand patients)** | | | | |
| --- | --- | --- | --- | --- | --- |
|  | **20%** | **40%** | **60%** | **80%** | **90%** |
| **Age** | 11.6 (11.3-11.9) | 23.3 (22.9-23.6) | 23.1 (22.7-23.5) | 11.8 (11.4-12.2) | 5.6 (5.3-5.8) |
| **Pure SOFA** | 6.7 (6.3-7.0) | 13.3 (12.9-13.6) | 13.4 (13.0-13.8) | 6.8 (6.5-7.2) | 3.1 (2.8-3.4) |
| **New York, ‘15** | 5.0 (4.6-5.4) | 10.5 (10.1-10.9) | 10.6 (10.2-11.0) | 5.2 (4.8-5.6) | 2.7 (2.4-3.0) |
| **Maryland, ‘21** | 3.3 (3.0-3.7) | 6.8 (6.4-7.3) | 6.7 (6.3-7.2) | 3.3 (2.9-3.7) | 1.5 (1.2-1.7) |
| **Colorado, 20** | 10.3 (10.0-10.6) | 21.1 (20.7-21.5) | 21.2 (20.8-21.6) | 10.5 (10.1-10.8) | 5.2 (4.9-5.5) |

**Table S5 C: Life-years Saved by Protocol at different Capacities.** Mean survival and 95% CI is reported for 250 Monte Carlo simulations at the indicated level of scarcity. All findings within and across capacity-levels are statistically significant to P < 0.05.

| **Protocol** | **Life-years Saved by Capacity (per thousand patients)** | | | | |
| --- | --- | --- | --- | --- | --- |
|  | **20%** | **40%** | **60%** | **80%** | **90%** |
| **Age** | 1372 (1357-1386) | 2739 (2725-2754) | 2731 (2719-2743) | 1375 (1365-1385) | 682 (675-688) |
| **Pure SOFA** | 339 (324-353) | 664 (649-680) | 679 (664-693) | 346 (332-360) | 163 (154-173) |
| **New York, ‘15** | 165 (150-180) | 323 (306-340) | 329 (313-346) | 164 (149-178) | 86 (76-97) |
| **Maryland, ‘21** | 173 (159-188) | 355 (339-372) | 332 (315-349) | 169 (155-183) | 81 (71-90) |
| **Colorado, ‘20** | 1070 (1055-1084) | 2149 (2135-2163) | 2143 (2130-2157) | 1077 (1068-1087) | 533 (525-542) |
