# Supplementary material for: Investigating ethical tradeoffs in crisis standards of care through simulation of ventilator allocation protocols
Source: PLoS One. 2024 Sep 12;19(9):e0300951. doi: 10.1371/journal.pone.0300951 (PMC11392394; doi:10.1371/journal.pone.0300951)
Supplement: S6 Appendix — (DOCX) [file pone.0300951.s006.docx]

## S6 Appendix: Patient Mix Sensitivity Analysis

Investigating Ethical Tradeoffs in Crisis Standards of Care through Simulation of Ventilator Allocation Protocols

Jonathan Herington, Jessica Shand, Jeanne Holden-Wiltse, Anthony Corbett, Richard Dees, Chin-Lin Ching, Margie Shaw, Xueya Cai, Martin Zand.

In a respiratory illness surge where CSC protocols are likely to be required, patients may overwhelmingly present with the dominant illness (in our dataset, COVID). To test the sensitivity of our findings to different patient mixes, we partitioned our patient encounter dataset into COVID+ and COVID- sub-populations. We then ran our simulation method to simulate a short-term wave of moderate scarcity (n = 300 patients at 50% capacity) with different distributions of patients drawn from our COVID+ and COVID- sub-datasets (0% to 100% COVID+). Each simulation was repeated 250 times, and the results are reported below. Taking the main dataset mix (~35%) as a reference, there was no significant difference in mean lives saved by patient mix, for any protocol except for the Pure SOFA protocol, which improved its performance markedly as the COVID+ rate increased, but still performing below both the Age and Colorado ’21 protocols.

**Table S6 A16: Lives Saved by Protocol at different mixes of COVID+ and COVID- patients.** Lives saved and 95% CI is reported for 250 Monte Carlo simulations at the indicated level of scarcity. † - Approximate % of COVID positive patients in main simulation dataset. *Statistically significant difference from COVID positive proportion (~35%) in main simulation dataset

| **Protocol** | **Lives Saved by % COVID+ Patients (per thousand patients)** | | | | | |  |
| --- | --- | --- | --- | --- | --- | --- | --- |
|  | **10%** | **35%^†^** | **70%** | **80%** | **90%** | **100%** | |
| **Age** | 29.6 (28.1-231.2) | 28.8 (27.2-30.3) | 29.9 (28.3-31.4) | 28.3 (26.7-30.0) | 28.3 (26.8-29.7) | 28.6 (27.2-30.1) | |
| **Pure SOFA** | 13.9 (12.4-15.4) | 16.7 (15.2-18.2) | **21.3* (19.8-22.8)** | **21.7* (20.2-23.2)** | **23.4* (21.8-25.1)** | **24.7* (23.3-26.1)** | |
| **New York, ‘15** | 14.8 (13.3-16.3) | 13.4 (11.8-14.9) | 12.6 (11.0-14.3) | 11.8 (10.0-13.5) | 13.1 (11.5-14.8) | 12.1 (10.6-13.5) | |
| **Maryland, ‘21** | 9.4 (7.7-11.0) | 7.0 (5.4-8.6) | 8.0 (6.4-9.7) | 6.9 (5.3-8.5) | 8.6 (7.0-10.1) | 7.2 (5.6-8.8) | |
| **Colorado, ‘20** | 25.1 (23.6-26.6) | 27.3 (25.7-29.0) | 27.7 (26.2-29.3) | 27.0 (25.5-28.6) | 28.2 (26.6-29.8) | 27.9 (26.4-29.4) | |

For mean life years saved, again taking the main dataset mix (~35%) as a reference, both the Age based protocol and the Colorado protocol exhibited statistically significant decreases in performance as the mix of COVID patients increased. No other protocols registered statistically significant differences in life years saved.

**Figure S6 A**: **Lives saved and life years saved at different mixes of COVID+ and COVID- patients.**


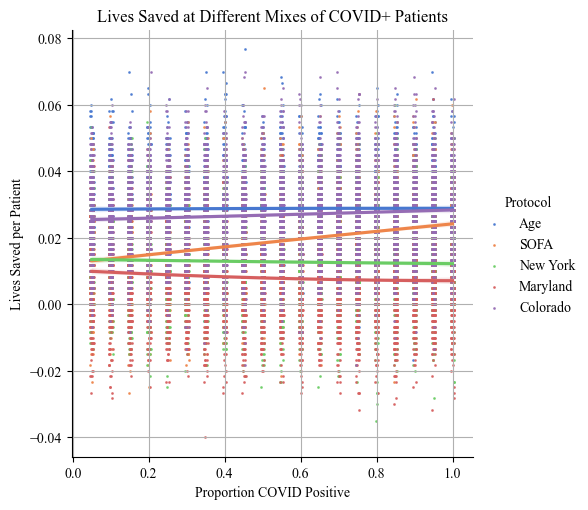

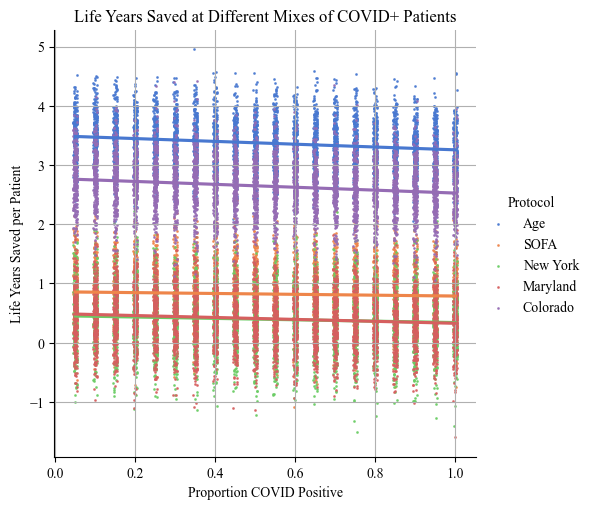


**Table S6 B:** **Life Years Saved by Protocol at different mixes of COVID+ and COVID- patients.** Life years saved and 95% CI is reported for 250 Monte Carlo simulations at the indicated level of scarcity. † - Approximate % of COVID positive patients in main simulation dataset. *Statistically significant difference from COVID positive proportion (~35%) in main simulation dataset

| **Protocol** | **Life Years Saved by % COVID+ Patients (per thousand patients)** | | | | | |  |
| --- | --- | --- | --- | --- | --- | --- | --- |
|  | **10%** | **35%^†^** | **70%** | **80%** | **90%** | **100%** | |
| **Age** | **3515* (3465-3566)** | 3406 (3353-3459) | 3343 (3289-3397) | 3323 (3270-3377) | **3253* (3204-3302)** | **3231* (3138-3280)** | |
| **Pure SOFA** | 828 (766-891) | 782 (718-845) | 824 (764-884) | 808 (750-867) | 773 (713-833) | 795 (738-851) | |
| **New York ‘15** | 521 (457-584) | 420 (358-483) | 369 (302-435) | 351 (290-413) | 355 (288-421) | 351 (295-408) | |
| **Maryland ‘21** | 471 (410-533) | 378 (312-443) | 396 (335-457) | 341 (282-400) | 332 (273-392) | 323 (261-386) | |
| **Colorado ‘20** | 2762 (2706-2817) | 2737 (2678-2796) | **2618* (2562-2674)** | **2568* (2515-2621)** | **2558* (2502-2615)** | **2547* (2493-2602)** | |
