# Supplementary material for: Investigating ethical tradeoffs in crisis standards of care through simulation of ventilator allocation protocols
Source: PLoS One. 2024 Sep 12;19(9):e0300951. doi: 10.1371/journal.pone.0300951 (PMC11392394; doi:10.1371/journal.pone.0300951)
Supplement: Attachment — Submitted filename: REPLY - Herington Sim Vent Allocaton.docx [file pone.0300951.s007.docx]

We thank you, the editors, and the three reviewers for your insightful comments on our submission entitled *"Investigating Ethical Tradeoffs in Crisis Standards of Care through Simulation of Ventilator Allocation Protocols."* (*PONE-D-24-08864*)

We have responded to each of the substantive criticisms and suggestions and think that the article is immensely improved. In particular, prompted by Reviewer 1 and 2’s comments we uncovered some errors in our implementation of the Colorado and the Bhavani/Maryland protocols. This necessitated re-simulating those protocols. While this did change the results for both revised protocols, the overall conclusions of the paper remain the same.

Nonetheless, we want to highlight to the editors and reviewers some of the major changes to the paper that they may wish to pay close attention to:

(1) We have removed reference to the “Bhavani” protocol and replaced it with a “Maryland ‘21” protocol that is very close to Bhavani multi-principle protocol except that it does not use age as a tiebreaker. This protocol was published in Chest in 2021 by a group of Maryland hospitals as their preferred set of standards. In order to correctly implement the Maryland ’21 protocol, we performed an extensive literature search to find work which associates Elixhauser scores with 1 year mortality. Of the limited work available, we were able to identify recent paper by Mehta et al (ref 19 in text), that identifies metastatic cancer as the Elix comorbidity with the highest odds ratio for 1 year mortality. We then used the van Walraven weight for metastatic cancer (“12”) as our threshold score for individuals with a severe comorbidity.
(2) We have also renamed the “Colorado” protocol to “Colorado ‘20” and clarified that it refers to the original April 2020 state plan, rather than the updated (creatinine sub-score adjusted) 2022 plan. We also re-wrote the algorithm which implements the Colorado ’20 modified Charlson Score to include the required points for age groups over 50 yrs. We emphasize in our discussion of the Colorado protocol that it is “age sensitive”.
(3) We ran an additional sensitivity analysis using different mixes of COVID+ patients in response to concerns that our patient population (~35% COVID+) would not accurately reflect the mix of patients during a COVID surge. As we report in the Disucssion, and the supplementary material, for almost all protocols there was no significant difference in lives saved /life years saved for different patient mixes. Only the Pure SOFA protocol shows a significant difference, with a small effect size.

After rectifying these oversights, we re-ran all simulations, sensitivity analyses and data analysis. This resulted in some major changes to the individual level results for the Colorado and Maryland/Bhavani protocol, but no major changes to the overall conclusions of the paper.

In the following table, we explain our changes in response to each reviewer comment. We have attached both clean and a ‘redlined’ version of the paper. We hope it aids in your review.

***Editors****: Please remove any funding-related text from the manuscript and let us know how you would like to update your Funding Statement. Currently, your Funding Statement reads as follows:*

*"JH, JHW, AC, and MZ received funding through the University of Rochester CTSA award number UL1 TR002001 from the National Center for Advancing Translational Sciences of the National Institutes of Health. The funder played no role in the study design, data collection and analysis, decision to publish or preparation of the manuscript."*

*Please include your amended statements within your cover letter; we will change the online submission form on your behalf.*

We have removed the funding-related text from the manuscript. The current funding statement is correct.

***Editors:*** *Please include your full ethics statement in the ‘Methods’ section of your manuscript file. In your statement, please include the full name of the IRB or ethics committee who approved or waived your study, as well as whether or not you obtained informed written or verbal consent. If consent was waived for your study, please include this information in your statement as well.*

We have included the following (p4, ln93-96):

*Our study involved secondary re-use of a de-identified version of this dataset. It was deemed by the University of Rochester Research Subject Review Board as exempt from review and a waiver of informed consent was granted. The dataset was fully anonymized by an honest broker before being accessed by the study team on the 15th of January 2022.*

***Editors:*** *Please upload a copy of Supporting Information Figure/Table/etc. Fig S7-S10 and Table S8-S11 which you refer to in your text on page 19.*

We have uploaded the supporting information as six .docx files, Appendix S1-S6 and generated appropriate references in the main text. Please let us know whether PLOS would prefer that we upload each figure and table within those appendices as a separate file.

***Editors:*** *Please review your reference list to ensure that it is complete and correct. If you have cited papers that have been retracted, please include the rationale for doing so in the manuscript text, or remove these references and replace them with relevant current references. Any changes to the reference list should be mentioned in the rebuttal letter that accompanies your revised manuscript. If you need to cite a retracted article, indicate the article’s retracted status in the References list and also include a citation and full reference for the retraction notice*

No retracted papers have been cited. All references are complete to the best of our knowledge.

**Reviewer 1:** 1. *More details on the SOFA score calculation should be added to the methods. Were the SOFA score components calculated automatically by the EHR software or by the investigators from the underlying EHR data? Was the original 1997 definition of SOFA used?*

We have added the following text to the methods:

“For each patient, the data included their ventilator status, their time on ventilator in days, and their sequential organ failure (SOFA) score, as calculated by the EMR using the original 1997 definition(23). The EMR calculated SOFA as the sum of the maximum score of each of the six SOFA sub-scores in the past 24 hours. Missing values relevant to SOFA score calculation within the first 24 hours of ventilation were assumed to be normal (rather than imputed), aligning with common practice and the necessity for timely data in a resource shortage.(11,24)”

**Reviewer 1:** 2. *When exactly was the SOFA score calculated? Was the worst value of each SOFA component in the first 24 hours following the initiation of ventilation used to calculate the total SOFA score? The discussion says the study only used “SOFA scores collected at point of intubation”, which implies this calculation was happening using data from the 24 hours prior to intubation. This needs to be clarified in the methods.*

Alongside the above text which clarifies the way in which SOFA was calculated, we provided the following additional text to clarify what the SOFA at the point of initial intubation relied upon.

“We also received the SOFA score present in the EMR at the point of intubation, which is calculated using data collected in the 24 hours prior to intubation.”

**Reviewer 1:** 3. *320 patients were excluded because they lacked lab results for SOFA, but earlier missing SOFA values were “assumed to be normal”.*

These excluded admissions lacked a SOFA score calculated by the EHR (at least at the point of intubation). We distinguish these from encounters that possess a SOFA calculated by the EMR, which may or may not have missing sub-score values.

We have updated the text to include:

“Of these, 897 admissions (19.5%) were excluded because they (i) lacked a SOFA score associated with their initial intubation (n=320), ….”

**Reviewer 1:** *4. What does “an unclear pattern of admissions and intubations (n = 332)” mean exactly?*

We agree that this is a little vague. In our de-identified dataset, as provided to us by the data broker, we had some admissions with multiple intubations (and associated max SOFA scores) but no clear way to identify the first or precipitating intubation. We decided to remove these encounters from the simulation dataset in order to ensure that each allocation decision was an initial intubation rather than a mix of initial intubations and re-intubation after trial extubation.

We have added the following text to clarify:

“The patient …. (iii) had multiple intubations within a single admission, but no indication of which SOFA scores were associated with initial intubations or re-intubation after trial extubation. We decided to remove this last set of encounters from the simulation dataset in order to ensure that each allocation decision was an initial intubation rather than a mix of initial intubations and re-intubation after trial extubation.”

”

**Reviewer 1:** *5. I would not recommend referring to the SOFA plus comorbidity with age tie-breaker as “the Bhavani protocol” throughout the paper. This protocol was not explicitly endorsed by Bhavani or the other authors of the manuscript and seems to have been designed to represent several different state plans (e.g., Maryland, Pennsylvania).*

Thank you for this suggestion, which prompted a reflection on which protocols we should simulate, and a finegrained review of the code-base to ensure our coded scores aligned with the reported protocols. During this review, we uncovered some errors which we detail below:

(1) Our naming convention for the Bhavani and Colorado protocols was likely too ambiguous – since there has been a shifting set of state standards and proposals throughout the pandemic.
(2) Our “Bhavani” and “Colorado” protocols were coded to use the *raw* SOFA score (range: 0, 23), rather than the SOFA Bands described for each protocol in the methods section. As we originally noted in the discussion (p13, ln 235) this Raw SOFA + Comorbidity model likely improves performance since it generates a much finer grained distribution of patients than SOFA bands.
(3) Our “Colorado” protocol did not include the adjustments for Age >50 that are included in the 2020 Colorado protocol. Instead, it used the list of comorbidities in the 2020 Colorado protocol to identify comorbidity bands (1-4) that were added to the SOFA score.
(4) Our implementation of the “Bhavani” multiprinciple protocol identified patients with “severe comorbidities” as all those patients with a Elixhauser (acute van Walraven) score of ≤3. This was selected based on our initial reading of the description of the Bhavani “multi-principle” protocol. In correspondence with one of the authors it became clear that this was the incorrect interpretation, and that the threshold was the 90% quantile of Elixhauser scores in their dataset.

Our goal in picking the New York, Colorado and “Bhavani” protocols was to identify protocols that used (i) SOFA tiers only, (ii) SOFA + comorbidity, and (iii) SOFA + comorbidity + age. Given that goal, and the errors described above, we have done the following:

(1) We have removed reference to the “Bhavani” protocol and replaced it with a “Maryland ‘21” protocol that is very close to Bhavani multi-principle protocol except that it does not use age as a tiebreaker. This protocol was published in Chest in 2021 by a group of Maryland hospitals as their preferred set of standards. We have also renamed the “Colorado” protocol to “Colorado ‘20” and clarified that it refers to the original April 2020 state plan, rather than the updated (creatinine sub-score adjusted) 2022 plan. It stands in as an “SOFA+comorbidity” protocol.
(2) and (3): We re-wrote our scoring algorithm to correctly implement the SOFA bands described in the published versions of both the Colorado ’20 and the Maryland ’21 protocols. We also re-wrote the algorithm which implements the Colorado ’20 modified Charlson Score to include the required points for different age groups over 50 yrs. The “Colorado ‘20” protocol thus stands in as our “SOFA+Comorbidity+Age protocol”
(4) We performed an extensive literature search to find work which associates Elixhauser scores with 1 year mortality. Of the limited work available, we were able to identify recent paper by Mehta et al (ref 19 in text), that identifies metastatic cancer as the Elix comorbidity with the highest odds ratio for 1 year mortality. We then used the van Walraven weight for metastatic cancer (“12”) as our threshold score for individuals with a severe comorbidity.

After rectifying these oversights, we re-ran all simulations, sensitivity analyses and data analysis. This resulted in some major changes to the individual level results for the Colorado and Maryland/Bhavani protocol, but no major changes to the overall conclusions of the paper.

**Reviewer 1:** *6. What was the etiology of respiratory failure requiring mechanical ventilation in the 66% of patients who did not have COVID? This information should be added to Table 1.*

Regrettably, in our present dataset, we cannot reliably identify the condition which precipitated intubation. The available dataset does not contain a “primary” or “principal” flag for the set of ICD-10-CM codes for each admission, and hence we only have an overall summary of the patient’s diagnoses for each admission (including chronic/underlying conditions).

As a proxy, we generated a list of all acute ICD-10-CM codes (identified using the HCUP Chronic Condition Indicator Refined, v 2024.1), for all patients in the COVID negative group. This produced a list of 3316 acute codes for 2430 covid negative encounters. While these counts are not mutually exclusive, they provide a window into the distribution of conditions in our ventilated, COVID negative population. We then aggregated these acutes diagnostic codes under broad clinical classifications (using the Clinical Classifications Software Refined, v2024.1). We report those results in Supplementary Table S8

CCSR Categories for Acute ICD-10-CM codes in our Covid negative population.

| **CCSR Category Description** | **Count** | **% of Codes** |
| --- | --- | --- |
| Respiratory signs and symptoms | 412 | 12.4% |
| Nervous system signs and symptoms | 194 | 5.9% |
| Cardiac arrest and ventricular fibrillation | 152 | 4.6% |
| Abnormal findings without diagnosis | 140 | 4.2% |
| Shock | 133 | 4.0% |
| Septicemia | 129 | 3.9% |
| Respiratory failure; insufficiency; arrest | 122 | 3.7% |
| Fluid and electrolyte disorders | 106 | 3.2% |
| Nonspecific chest pain | 100 | 3.0% |
| Circulatory signs and symptoms | 93 | 2.8% |
| Acute and unspecified renal failure | 82 | 2.5% |
| Pericarditis and pericardial disease | 72 | 2.2% |
| Other unspecified injury | 67 | 2.0% |
| Cardiac dysrhythmias | 58 | 1.7% |
| Abdominal pain and other digestive/abdomen signs and symptoms | 57 | 1.7% |
| **TOTAL** | **2430** | **100%** |

**Reviewer 1:** *7. Related to the previous comment, the patient population during a crisis scenario would likely differ from the general population who required mechanical ventilation from 4/2020- 5/2021. For example, during an overwhelming COVID-19 surge, patients who required mechanical ventilation following an elective surgery would not be included and the cohort would likely be mostly COVID +. I recommend the authors run sensitivity analyses with sub-populations for specific crisis scenarios (e.g. 90% COVID + and no elective surgery patients).*

We thank the reviewer for this comment, which spurred us to perform an additional sensitivity analysis which we report in Supplemental Data Appendix H. In this analysis, we partitioned our patient encounter dataset into COVID+ and COVID- sub-populations. We then ran our simulation method to simulate a sustained wave of moderate scarcity (n = 300 patients at 50% capacity) with different distributions of patients drawn from our COVID+ and COVID- sub-datasets (0% to 100% COVID+ at 5% increments). Each simulation was repeated 1000 times, and the results are reported below.

While there is some statistically significant variation in mean lives saved and/or life years saved by patient mix for the pure SOFA protocol, no other protocol exhibited significant differences by patient mix.

Regrettably, in our present dataset, we cannot reliably identify patients who were intubated in the course of elective procedures. Nonetheless we were able to identify 124 admissions with ICD-10-CMs for “preprocedural examinations” (Z01.81*) that upon review highly correlate with suspected elective procedures such as solid organ transplant. Excluding these from our sensitivity analysis has a marginal effect on results, and so we have not reported that work. Instead we simply note in the limitations, an inability to identify elective procedures.

**Reviewer 1:** *8. Did the authors race-adjust life-expectancy and life-years in Table 4? I would argue this choice normalizes racial disparities in life-expectancy which hopefully will improve over time with efforts to address structural inequity in healthcare. So I recommend these statistics be re-calculated for age-alone.*

The “life years saved” metric is calculated as the difference between expected life years for a given capacity level (i.e sum of life expectancy of those discharged at baseline, multiplied by beds per patient) and life years for each protocol (i.e. sum of life expectancy of those who survived in simulated protocol run). Life expectancy (if discharged) for each patient is determined by (i) lookup by age, sex and race/ethnicity of NVSS life expectancy tables, and (ii) adjustment by comorbidity band (none, low, high), age, sex and race/ethnicity using the modifiers calculated by Cho et. al., 2013.

Our goal with this method is to control for significant racial disparities in life expectancy (and survival) in the baseline dataset. This allows us to isolate the marginal effect on racial disparities of each protocol, in order to provide a comparison that is less sensitive to our dataset’s underlying distribution of survival and life expectancy in each racial sub-group.

We do agree, however, that this way of calculating baseline life expectancy accepts as a background condition the racial disparities in life expectancy that are reported in the NVSS. To help elucidate these disparities (and others) in our dataset we have created an additional supplementary table Table S6 that describes the distribution of various features (age, sex, COVID status, Charlson, Elixhauser, life expectancy, and comorbidity-adjusted life expectancy) for the three race/ethnicity sub-populations. We note in the discussion section (p16) that the distribution of health between racial groups in our dataset does not reflect national health disparities. We note that this is likely because (i) our white sub-population is significantly older than our Black and Hispanic sub-populations, and (ii) there is possible population bias in URMC’s patient pop, whereby we may receive comparatively healthier (and higher income, younger) Black and Hispanic patients than other regional health centers.

**Reviewer 1:** *9. The updated Colorado guidelines currently include a correction to the renal SOFA score component in an attempt to debias SOFA. I recommended adding this to the simulation results.*

Regrettably, our present de-identified dataset does not include the underlying SOFA-sub scores. We have noted that our simulation utilizes the original April 2020 Colorado guidelines without Creatinine sub-score correction.

**Reviewer 2:** *Although the most optimal way to stratify patients during periods of CSC remains unclear, this paper advocates that SOFA alone may not be enough, and that age should be included in some way. It's important to note that the Colorado system utilizes the Charlson comorbidity Index, and this index contains age as one of the components in its calculation with significant weight.*

Thank you for this nudge. As we note in our reply to Reviewer 1, in our simulation of the Colorado protocol in our original submission, we failed to include the age-weights in the modified Charlson Score. We have re-simulated the Colorado protocol with an accurate rendering of the age weights, and re-emphasized the inclusion of age in our discussion of the Colorado protocol.

**Reviewer 2:** *I also had a question about the relatively low percentage of COVID cases. During this crisis time frame, most hospitals were overwhelmed with COVID cases, typically shutting down ORs except for emergency cases, etc. It seems odd that only 34% of the critically ill cases would be secondary to COVID. The overall survival between COVID and non COVID was similar, but was there a further subgroup analysis?*

*Please see our response to Reviewer 1’s similar concern. We performed a sensitivity analysis by re-simulating all protocols using different mixes of COVID+ and COVID- patients, and identified very little variation except for a small upward trend in the performance of the Pure SOFA protocol at high levels of COVID+ patients.*
